# *Vibrio cholerae* Evades Neutrophil Extracellular Traps by the Activity of Two Extracellular Nucleases

**DOI:** 10.1371/journal.ppat.1003614

**Published:** 2013-09-05

**Authors:** Andrea Seper, Ava Hosseinzadeh, Gregor Gorkiewicz, Sabine Lichtenegger, Sandro Roier, Deborah R. Leitner, Marc Röhm, Andreas Grutsch, Joachim Reidl, Constantin F. Urban, Stefan Schild

**Affiliations:** 1 Institute of Molecular Biosciences, University of Graz, Graz, Austria; 2 Department of Molecular Biology, Umeå University, Umeå, Sweden; 3 Laboratory for Molecular Infection Medicine Sweden (MIMS), Umeå University, Umeå, Sweden; 4 Umeå Centre for Microbial Research, Umeå University, Umeå, Sweden; 5 Institute of Pathology, Medical University of Graz, Graz, Austria; 6 Department of Clinical Microbiology, Umeå University, Umeå, Sweden; Max-Planck Institute for Infection Biology, Germany

## Abstract

The Gram negative bacterium *Vibrio cholerae* is the causative agent of the secretory diarrheal disease cholera, which has traditionally been classified as a noninflammatory disease. However, several recent reports suggest that a *V. cholerae* infection induces an inflammatory response in the gastrointestinal tract indicated by recruitment of innate immune cells and increase of inflammatory cytokines. In this study, we describe a colonization defect of a double extracellular nuclease *V. cholerae* mutant in immunocompetent mice, which is not evident in neutropenic mice. Intrigued by this observation, we investigated the impact of neutrophils, as a central part of the innate immune system, on the pathogen *V. cholerae* in more detail. Our results demonstrate that *V. cholerae* induces formation of neutrophil extracellular traps (NETs) upon contact with neutrophils, while *V. cholerae* in return induces the two extracellular nucleases upon presence of NETs. We show that the *V. cholerae* wild type rapidly degrades the DNA component of the NETs by the combined activity of the two extracellular nucleases Dns and Xds. In contrast, NETs exhibit prolonged stability in presence of the double nuclease mutant. Finally, we demonstrate that Dns and Xds mediate evasion of *V. cholerae* from NETs and lower the susceptibility for extracellular killing in the presence of NETs. This report provides a first comprehensive characterization of the interplay between neutrophils and *V. cholerae* along with new evidence that the innate immune response impacts the colonization of *V. cholerae* in vivo. A limitation of this study is an inability for technical and physiological reasons to visualize intact NETs in the intestinal lumen of infected mice, but we can hypothesize that extracellular nuclease production by *V. cholerae* may enhance survival fitness of the pathogen through NET degradation.

## Author Summary

Although several reports describe an inflammatory component of the diarrheal disease cholera, the innate immune response to *V. cholerae* and its impact on the pathogenesis of the disease is poorly understood. In the present study we can link the presence of host neutrophils with a colonization defect of a *V. cholerae* mutant deleted for both extracellular nucleases, Dns and Xds. Neutrophils can be seen as a first line of defense of the innate immunity and can effectively entrap and kill pathogens in neutrophil extracellular traps (NETs). Herein we show for the first time that *V. cholerae* induces NET formation, but effectively uses its two extracellular nucleases to degrade NETs and evade from this innate immunity weapons. Interestingly, we recently characterized the two extracellular nucleases as modulators of extracellular DNA during biofilm formation, which is rather associated with environmental lifestyle of this facultative human pathogen in aquatic ecosystems. Thus, *V. cholerae* seems to utilize the activity of the extracellular nucleases under both stages of its lifecycle, inside the host as a defense mechanism against NETs and during biofilm formation in the environment.

## Introduction

The Gram negative facultative human pathogen *Vibrio cholerae* is the causative agent of cholera, which is defined as an acute, secretory diarrheal disease. Today, the global burden of cholera is estimated to reach several million cases per year, with the majority located in the endemic areas of Africa and Asia [1]. However, explosive outbreaks facilitated by natural disasters, high population density and poor sanitation can occur worldwide as recently demonstrated by the cholera epidemic in Haiti, where cholera cases have not been reported before 2010 [2].

The lifecycle of clinically relevant *V. cholerae* serogroup O1 and O139 is marked by two distinct phases. *V. cholerae* is not only a pathogen of the human gastrointestinal tract, but also a natural inhabitant of aquatic ecosystems, which serve as important reservoirs during periods between epidemics. Biofilm formation on chitinous surfaces provided by zoo- and phytoplankton as well as entry into a viable but non-culturable state are thought to be important for persistence within these nutrient limited environments [3], [4]. Infection usually starts with the oral ingestion of *V. cholerae* with contaminated food or water. The infectious dose is quite high and ranges from 10^6^ to 10^8^ depending on the acidity in the stomach and overall health of the human being [5], [6]. After passage through the stomach, *V. cholerae* reaches the small bowel, its primary site of colonization, and induces virulence factors such as the toxin coregulated pilus and the cholera toxin. Incubation periods from 12 h up to several days have been described, before the first symptoms can be recognized [5], [6]. Due to the activity of the cholera toxin, the patient develops a massive watery diarrhea with volumes of up to 20 l stool per day, which can rapidly lead to life threatening dehydration, hypotensive shock and organ failure. Without treatment the case-fatality rate for severe cholera can exceed 70% [5]. *V. cholerae* leaves the host in a transient phenotype called hyperinfectivity, which is characterized by a infectious dose 10 to 100-fold lower compared to in vitro-grown bacteria [7]. In addition, *V. cholerae* exhibits an exceptional growth rate in the gastrointestinal tract and exits the human host at relatively high numbers of up to 10^8^ CFU per ml patient stool with the onset of the diarrhea [6], [8]. These observations provide some explanation for the rapid transmission and explosive spread of cholera during outbreaks.

In general, cholera is still considered to be rather a noninflammatory secretory disease. However, microscopical studies conducted by Mathan and coworkers in 1995 revealed an activation and increase in inflammatory cells in the gut of cholera patients [9]. Subsequent studies demonstrated a broad upregulation of inflammatory cytokines as well as recruitment of polymorphonuclear neutrophils (PMNs) in the gastrointestinal tract of patients during the acute phase of cholera [10]–[12]. Signals stimulating the inflammatory response seem to be diverse. Recent studies demonstrated that *Vibrio* flagellins activate the inflammatory response via TLR5, lipopolysaccharide (LPS) is recognized through TLR4 and lipoproteins by TLR1/2 [13]–[15]. Interestingly, *V. cholerae* toxins seem to have some anti-inflammatory activity. In vitro studies demonstrated that pretreatment with cholera toxin suppresses the induction of cytokines in LPS-stimulated macrophages [16]. These results provide some explanation for the observed side effects including a pronounced inflammatory response in human volunteers vaccinated with live-attenuated oral nontoxigenic *V. cholerae* strains, which were deleted for the cholera toxin [17], [18]. In addition, it was recently suggested that the cytolytic and proteolytic activity of the accessory toxins could affect innate immune cells, including neutrophils, and may therefore facilitate prolonged colonization in the host [19], [20].

A first line of defense and central part of the human innate immunity are neutrophils, which are recruited to the site of infection by chemokines secreted from macrophages or local cells upon contact with microbial pathogens. Besides phagocytic activity, activated neutrophils can release NETs, which are composed of decondensed chromatin associated with granular and cytoplasmic proteins [21], [22]. These NETs can effectively entrap and promote extracellular killing of bacteria, most likely due to their high serine protease content [21]. Extracellular nucleases of the Gram positive pathogens *Staphylococcus aureus*, *Streptococcus pneumoniae* and group A *Streptococcus* have been reported to play a role in the evasion of neutrophil defenses via degradation of the NET-backbone, enabling the liberation of the bacteria from NETs [23]–[25]. Interestingly, *V. cholerae* encodes two extracellular nucleases Dns and Xds, which exhibit endo- and exonuclease activity, respectively. Recently, we identified *xds* as a gene induced at a late stage of infection [26]. While a minor fraction of these late genes were required for maintenance of colonization, most of them were found to facilitate the transition fitness of *V. cholerae* from the host into the aquatic lifestyle [26]. Thus, we initially investigated the physiological function of the two extracellular nucleases Dns and Xds of *V. cholerae* during biofilm formation and characterized them as modulators for extracellular DNA in the *V. cholerae* biofilm matrix [27]. Nevertheless, the induction in vivo prompted us to investigate whether the nucleases also play a role during infection and provide a mechanism against NETs.

In the present study, we show that the extracellular nucleases increase the colonization fitness in immunocompetent mice, but are not required for colonization in mice depleted for neutrophils (neutropenic mice). Based on this observation, we comprehensively characterized the interplay between neutrophils and *V. cholerae* and demonstrate that the extracellular nucleases are essential for NET degradation, mediate evasion from NETs and thereby increase survival upon contact with neutrophils.

## Results

### Extracellular nucleases of *V. cholerae* facilitate intestinal colonization in immunocompetent but not neutropenic mice

To determine whether there is a difference in the colonization efficiency of wild type and double nuclease mutant, we conducted single strain infections using an adult mouse model according to Nygren et al. [28]. The streptomycin-treated adult mouse model was chosen over the otherwise frequently used infant mouse model, because infant mice have been reported to exhibit an innate immunity with impaired cytokine response, i.e. interleukin IL-1 and IL-6 [29]–[31]. Furthermore, the streptomycin-treated adult mouse model allows a stable colonization of *V. cholerae* for at least 72 h, which offers the possibility to investigate the impact of the innate immune response on maintenance of the infection [28]. Stable colonization with *V. cholerae* in the streptomycin-treated adult mouse is limited to the cecum and colon, while the pathogen is rapidly cleared from the small intestine. That is why, the results of the current study were obtained from cecal or colonic tissues. Longer persistence of *V. cholerae* in the small intestine requires the additional use of ketamine anesthesia [32]. Unfortunately, ketamine has been reported to affect neutrophil activation and recruitment [33]–[36]. Thus, ketamine treatment might have masked innate immune mechanisms relevant for this study and was not applied.

**Figure 1:**
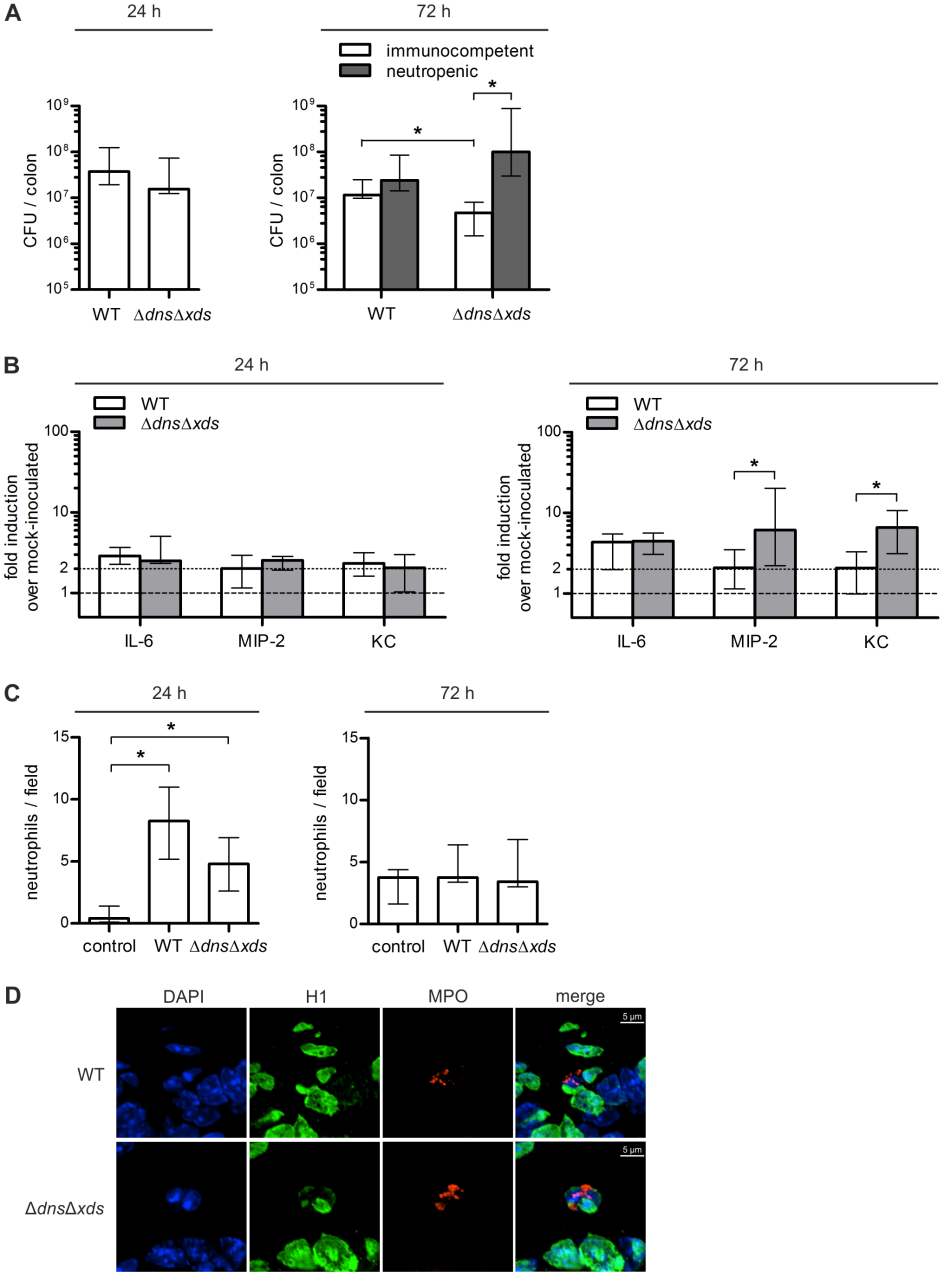
Intestinal colonization, inflammatory gene expression and neutrophil infiltration. **A**. Shown are the recovered CFU per colon from immunocompetent (open bars) or neutropenic (gray bars) C57BL/6 mice infected with *V. cholerae* WT or Δ*dns*Δ*xds* mutant. Eight to ten weeks old streptomycin-treated mice were inoculated with the respective *V. cholerae* strain (7×10^9^ to 10^10^ CFU per mouse). At 24 h or 72 h post infection, the colons were collected, homogenized in LB medium and plated for CFU counting. Shown are medians of the recovered CFU for each data set (n = 5 for 24 h and n≥7 for 72 h). The error bars indicate the interquartile range. Significant differences between the data sets are marked by asterisks (*P*<0.05; Mann-Whitney U Test). **B**. Shown is the induction of inflammatory gene expression upon *V. cholerae* infection. Eight to ten weeks old streptomycin-treated C57BL/6 mice were inoculated with *V. cholerae* WT, Δ*dns*Δ*xds* mutant or left uninfected for mock-inoculated controls. At 24 h or 72 h post infection, the proximal 1 cm of the ascending colon was collected, RNA was extracted, reverse transcribed to cDNA and used as template for qRT-PCR analysis of the indicated genes. Gene expression was normalized to the housekeeping gene 36B4. Shown are median gene expression levels compared to mock-inoculated controls (indicated by the dashed line at 1) for each data set (n = 5 for 24 h and n≥7 for 72 h). The error bars indicate the interquartile range. The dotted line indicates a 2-fold upregulation compared to the mock-inoculated control mice. Significant differences between the data sets are marked by asterisks (*P*<0.05; Mann-Whitney U Test). **C**. Infiltration of the epithelium with neutrophils was quantified by randomly counting fifty high-power fields (HPF, 400× magnification) in tissue sections of ceca of mice colonized for 24 h or 72 h with *V. cholerae* WT, Δ*dns*Δ*xds* mutant or mock-inoculated controls (n = 5 for 24 h and n≥7 for 72 h). The results are expressed as the median number of neutrophils per field. The error bars indicate the interquartile range. Significant differences between the data sets are marked by asterisks (*P*<0.05; Kruskal-Wallis test followed by post-hoc Dunn's multiple comparisons). **D**. Shown is a representative immunofluorescence staining of neutrophils in ceca from mice colonized for 24 h with *V. cholerae* WT or Δ*dns*Δ*xds* mutant. Neutrophils were visualized by indirect immunofluorescence using primary antibodies against histone H1 and neutrophil myeloperoxidase (MPO) and DNA was visualized with DAPI (blue channel). Alexa Fluor 488- and 568-conjugated secondary antibodies were used for visualization of histone H1 (green channel) and MPO (red channel), respectively. Pictures were taken with a Nikon C1 confocal microscope at 60× magnification. Maximum intensity projections from Z-stacks are shown.

C57BL/6 mice were inoculated intragastrically with *V. cholerae* wild type or the Δ*dns*Δ*xds* mutant and colonization was analyzed after 24 h and 72 h (Fig. 1A, open bars). Although all mice showed a considerable amount of recoverable CFU at both time points, the double nuclease mutant showed a tendency towards a slightly reduced colonization. This trend consolidated over time and resulted in a significantly reduced median colonization of the double nuclease mutant compared to wild type at 72 h (Fig. 1A, open bars).

Induction of inflammatory and regulatory cytokine and chemokine expression was assessed by qRT-PCR in tissue of *V. cholerae* infected mice and compared to streptomycin-treated, but uninfected control mice referred to as mock-inoculated controls from hereon (Fig. 1B). At 24 h the expression levels of IL-6, the macrophage inflammatory protein 2 alpha (MIP-2) and the keratinocyte derived cytokine (KC), representing a functional homolog of the human IL-8 [37], [38], were already 2-fold elevated in mice infected with *V. cholerae* wild type or Δ*dns*Δ*xds* mutant. At 72 h the inflammatory response was generally more apparent and characterized by 2 to 7-fold upregulated expression levels of the inflammatory markers in mice infected with *V. cholerae* wild type or Δ*dns*Δ*xds* mutant compared to the mock-inoculated control group. In concordance with the current literature, these data demonstrate an inflammatory response in the gastrointestinal tract upon infection with *V. cholerae* [10]–[12], [19]. Noteworthy, at 72 h distinct differences in the inflammatory response between wild type and Δ*dns*Δ*xds* mutant infected mice could be observed (Fig. 1B). While the induction of IL-6 was quite comparable in both infection groups, Δ*dns*Δ*xds* mutant infected mice exhibited a significant higher expression of MIP-2 and KC compared to wild type infected mice. MIP-2 (CXCL2) and KC (CXCL1) belong to the CXC chemokines, which are potent chemoattractants involved in neutrophil recruitment to the site of infection [39]–[42].

In further consistency with previous reports [9], [10], [43], [44], infiltration of neutrophils was detected for mice colonized with wild type and Δ*dns*Δ*xds* mutant by histological analysis of cecal tissue sections (Fig. 1C and S1). At 24 h post infection mice colonized with wild type and Δ*dns*Δ*xds* mutant exhibited a significant higher amount of neutrophils infiltrating the tissue compared to the mock-inoculated control group (Fig. 1C). Immunofluorescence staining of histone H1 and DNA along with myeloperoxidase (MPO), a marker for neutrophils [45], was used to support the neutrophil infiltration in the epithelium (Fig. 1D). At 72 h the effects became indistinct due to a notable increase of neutrophils in the mock-inoculated control group, which is most likely caused by side effects from the continuous treatment with antibiotics (Fig. 1C). Furthermore, the peak of neutrophil infiltration in mice colonized with *V. cholerae* for 72 h might have already passed. Thus, most of the neutrophils might have reached the intestinal lumen and underwent activation, while histological analysis allows only the quantification of the remaining neutrophils.

Based on these results, we hypothesized that the observed colonization defect of the double nuclease mutant at 72 h might result from an impaired evasion from neutrophils. Thus, colonization fitness of the wild type and the Δ*dns*Δ*xds* mutant was assayed in neutropenic mice, which were generated by treatment with Anti-Ly6G mAb prior to infection [46]. In the case of the infection with *V. cholerae* wild type almost comparable colonization levels in immunocompetent and neutropenic mice were observed (Fig. 1A, right panel). In contrast, the median colonization of the Δ*dns*Δ*xds* mutant in neutropenic mice was significantly increased by 10-fold compared to immunocompetent mice (Fig. 1A, right panel). These data suggest that the different colonization fitness of the double nuclease mutant in immunocompetent and neutropenic mice depends on the presence of neutrophils.

### *V. cholerae* induces NET formation and degrades NETs by the activity of two extracellular nucleases

**Figure 2:**
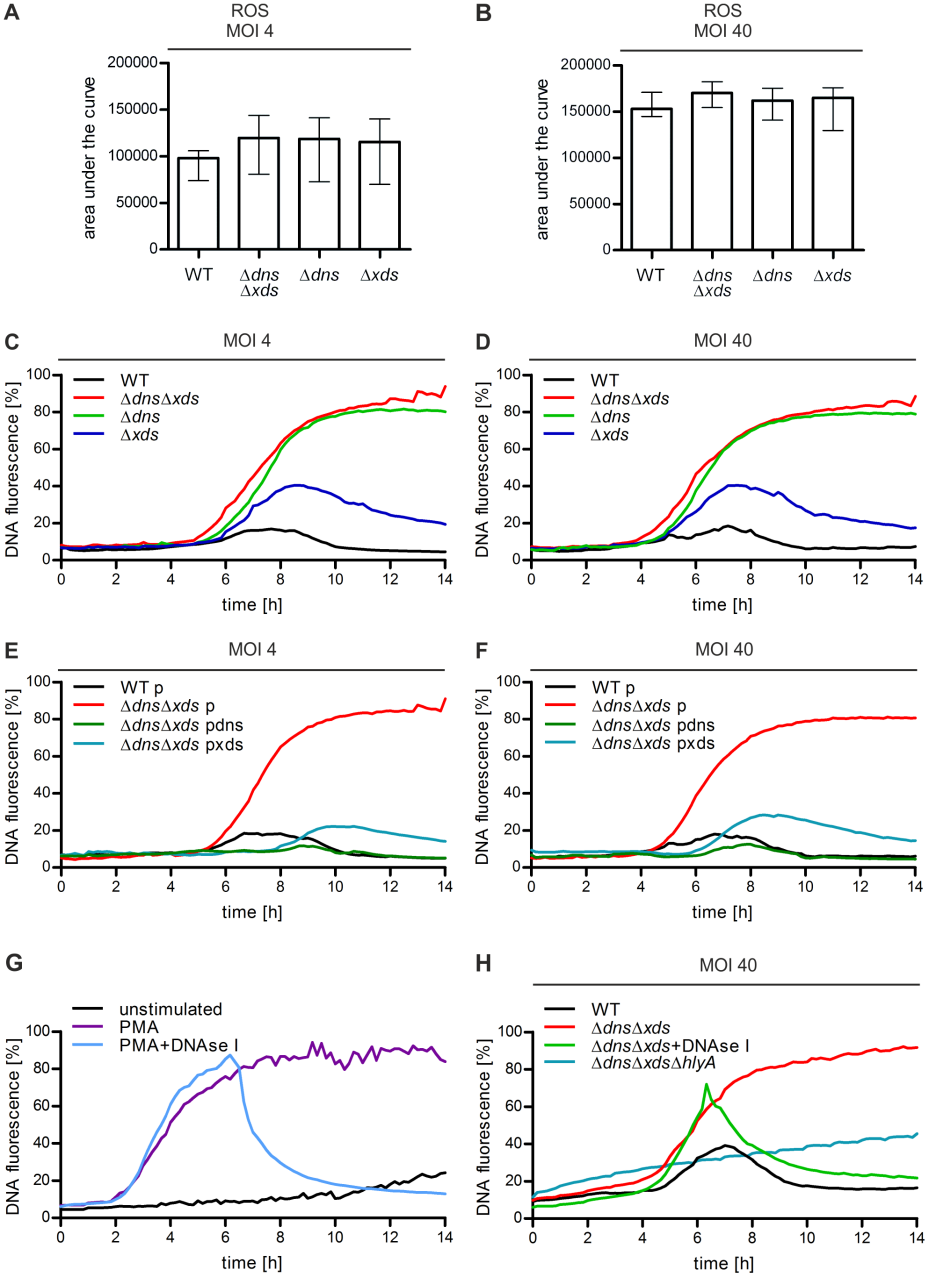
*V. cholerae* stimulates ROS production and NET formation in human neutrophils. **A–B**. ROS production by human neutrophils incubated with the indicated *V. cholerae* strains and MOI was measured by a luminometric assay. The y-axis shows the area under the curve representing the ROS production over 6 h. Shown are medians of at least six measurements out of three independent donors. The error bars represent the interquartile range. The ROS dynamics are available as supporting figure S2B and C. **C–H**. DNA release of neutrophils incubated with PMA or the respective *V. cholerae* strain and MOI. Untreated neutrophils served as an unstimulated control. Staining of DNA by the cell impermeant fluorescent DNA dye Sytox green was measured in 10 min intervals. Values are presented as percentage of DNA fluorescence compared with the Triton ×100 lysis control (100%) indicating NET formation, respectively. DNAse I was added after 6 h for the indicated data sets (+DNAse I). Shown are medians of at least six measurements out of three (C–F) or two (G and H) independent donors.

These in vivo results prompted us to investigated the direct interplay of neutrophils and *V. cholerae*, since the interaction between neutrophils and *V. cholerae* is largely uncharacterized. First, we analyzed the reactive oxygen species (ROS) production of neutrophils upon contact with *V. cholerae*, since the oxidative burst is a key feature for neutrophils, which is involved in signaling processes as well as microbial killing [45], [47]. ROS production of human neutrophils incubated with *V. cholerae* wild type, Δ*dns*Δ*xds*, Δ*dns* or Δ*xds* mutant was measured in a luminometric based plate assay over a time period of 6 h. As a positive control neutrophils were stimulated with phorbol myristate acetate (PMA), which is a potent, non-physiological activitor for ROS production. Unstimulated neutrophils served as negative control. As expected PMA-stimulated neutrophils showed pronounced ROS production, while no increase in ROS occurred in unstimulated neutrophils (Fig. S2A). A robust ROS production in a MOI dependent manner was observed for all *V. cholerae* strains tested (Fig. 2A and B), indicating that *V. cholerae* is recognized by human neutrophils. Furthermore, the peak of ROS production is reached at approximately 4 h (MOI 4) or at approximately 3 h (MOI 40) independent of the strains tested (Fig. S2B and C). Since the detected ROS levels and dynamics are comparable for wild type and nuclease mutants, the recognition and activation potential of neutrophils by *V. cholerae* is independent of the presence of the two extracellular nucleases. Interestingly, bacterial extracellular nucleases have been shown to degrade NETs, which are released upon microbial stimulation and represent chromatin decorated with antimicrobial proteins [25]. Moreover, ROS has been reported to be essential for NET formation [45], [47].

To test whether *V. cholerae* is able to induce NET formation, a fluorescent-based quantification of DNA released by neutrophils was performed. In detail, DNA was quantified using the cell impermeant fluorescent dye Sytox green, which only detects extracellular DNA or DNA not surrounded by an intact membrane. In the case of NET formation the plasma membrane of neutrophils gets ruptured and DNA decorated with antimicrobial peptides is released. Consequently, released DNA is stained by Sytox Green, which results in an increase in the fluorescent signal and is a well established technique to quantify NET formation [45]. Neutrophils were stimulated with wild type, Δ*dns*Δ*xds*, Δ*dns* or Δ*xds* mutant (Fig. 2C–F). Again, PMA-stimulated and unstimulated neutrophils served as positive and negative control, respectively (Fig. 2G). Release of DNA started approximately after 2 h for the PMA-stimulated neutrophils, while the fluorescent signal remained at very low levels in the unstimulated control. To confirm that the assay indeed measures DNA, Dnase I was added to neutrophils 6 h after PMA-stimulation (Fig. 2G). As expected the signal rapidly decreased due to the degradation of extracellular DNA. In the case of neutrophils stimulated with Δ*dns*Δ*xds* or Δ*dns* mutant the NET level increased over time after a lag-phase of 4 to 6 h and reached a plateau at approximately 80% of total DNA (Fig. 2C and D). Notably, wild type stimulated neutrophils showed only a slight increase in the NET level with the maximum at 20% followed by a decline. The single nuclease mutant Δ*xds* displayed an intermediate phenotype. An increase of the MOI had neither an effect on the general trends nor on the maximal level of NET formation, but resulted in a slightly shortened lag-phase (Fig. 2C and D). Again, addition of DNAse I to neutrophils stimulated with the double nuclease mutant after 6 h resulted in a rapid decrease of the fluorescence intensity confirming that the fluorescence signal reflects liberated DNA (Fig. 2H). As demonstrated above all *V. cholerae* strains tested showed similar induction of ROS-levels (Fig. 2A and B). Thus, the lower NET formation in the wild type or Δ*xds* cannot simply be explained by a lower level of neutrophil activation. Furthermore, complementation to wild type levels or even below could be achieved for the double or single nuclease mutants by expression of *dns* or *xds* in trans on a plasmid for both MOI doses tested (Fig. 2E and F, Fig. S3A and B). The vector control showed no effect on the observed phenotypes in all strains tested. In summary, reduced NET formation observed in this assay correlates with expression of the extracellular nucleases. Since the readout of this assay is based on the detection of the released DNA from neutrophils, the data suggests that the *V. cholerae* nucleases degrade NETs very efficiently.

In order to eliminate the possibility that the observed NET formation is strain specific, the assay was repeated in another *V. cholerae* clinical isolate including the respective nuclease mutants with essentially the same result (Fig. S3C and D). Recently, Valeva et al. reported that the *V. cholerae* cytolysin attacks the membrane of granulocytes and thereby stimulates ROS production, activates degranulation and release of elastase [48]. In order to test whether the cytolysin also affects liberation of DNA by neutrophils we constructed a triple mutant deleted for the cytolysin (HlyA) as well as for the two extracellular nucleases and measured the amount of released DNA by the fluorescence assay. Indeed, the amount of liberated DNA is reduced by 50% in the triple mutant compared to the double mutant (Fig. 2H). Thus, the cytolysin seems to be a potent stimulus for degranulation and DNA release. The residual activity in the triple mutant indicates additional stimuli of *V. cholerae*, which can induce NET formation.

**Figure 3:**
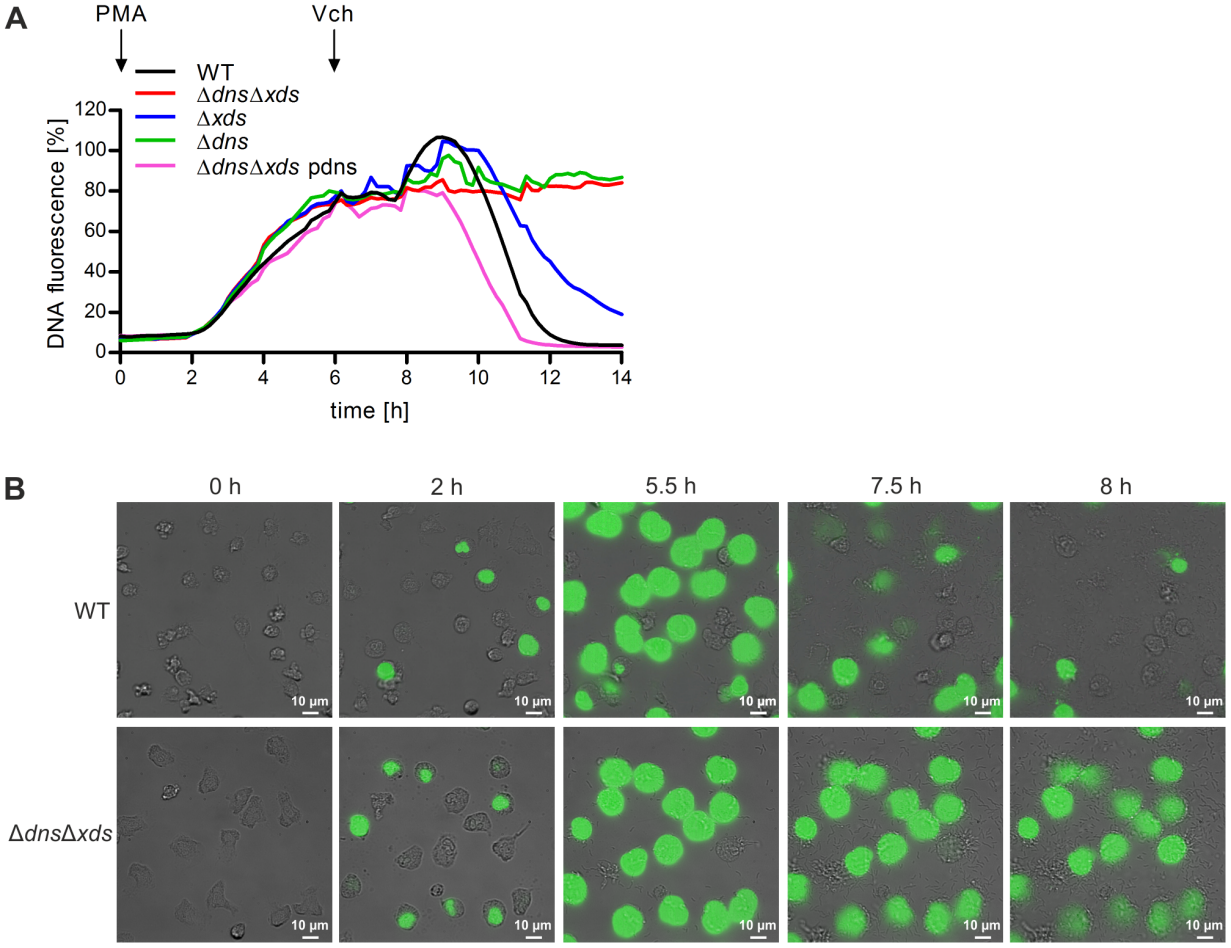
The two extracellular nucleases of *V. cholerae* are able to degrade NETs. **A**. DNA release of neutrophils was stimulated with PMA (indicated by the arrow “PMA”) and followed by incubation with the respective *V. cholerae* strain (MOI 40, time point of addition is indicated by the arrow “Vch”). Staining of DNA by the cell impermeant fluorescent DNA dye Sytox green was measured in 10 min intervals. Values are presented as percentage of DNA fluorescence compared with the Triton ×100 lysis control (100%) indicating NET formation, respectively. Shown are medians of at least six measurements out of three independent donors. **B**. Human neutrophils were stimulated with *V. cholerae* WT or Δ*dns*Δ*xds* mutant (MOI 4) in presence of the cell impermeant fluorescent DNA dye Sytox green and monitored by live cell imaging. Shown are images of the indicated time points. The complete movies are available as movie S1 (*V. cholerae* WT) and S2 (Δ*dns*Δ*xds* mutant) in the supporting information.

To confirm the hypothesis that Dns and Xds degrade DNA of NETs, we performed a degradation assay using the same fluorescent-based assay as for the NET quantification (Fig. 3A). In contrast to the previous assay, NET formation was stimulated with PMA prior to the addition of the wild type, Δ*dns*Δ*xds*, Δ*dns* or Δ*xds* mutant. This allowed a quantification of the degradation capacity of all strains on already formed NETs. In samples incubated with Δ*dns*Δ*xds* or Δ*dns* mutant no degradation was visible, while incubation with wild type or Δ*xds* resulted in pronounced degradation of the NETs within 8 h. Expression of *dns* in trans restored the degradation capacity of the double nuclease mutant even beyond wild type levels. In both assays presented in Fig. 2 and 3A, Δ*dns* shows more pronounced effects than Δ*xds*. Thus, the endonuclease Dns seems to be more efficient in degradation of NETs compared to the exonuclease Xds. However, the wild type exhibits the most prominent degradation capacity, which suggests that both nucleases act somewhat synergistically.

In addition, live cell microscopy was performed over a period of 8 h to monitor neutrophils stimulated with wild type or Δ*dns*Δ*xds* mutant in presence of the cell impermeant fluorescent dye Sytox green (Movie S1 and S2). This allowed temporal detection of NET formation as well as NET degradation on the cellular level by appearance and disappearance of a fluorescent signal. Images of selected time points along the experiment are provided in Fig. 3B. Within the first hours a similar progression of NET induction was monitored for wild type and Δ*dns*Δ*xds* mutant, reaching a maximum level of intensity at 5.5 h. From there the fluorescent signal remained quite stable in case of the Δ*dns*Δ*xds* mutant over the entire imaging period, which indicates no NET degradation. In contrast, the fluorescent signal steadily vanished in the presence of the wild type leaving only few remaining signals at the endpoint of the experiment. Taken together, these results demonstrate that wild type and Δ*dns*Δ*xds* mutant stimulate NET formation to an equal level, but only the wild type has the ability to degrade NETs by the activity of the two extracellular nucleases.

**Figure 4:**
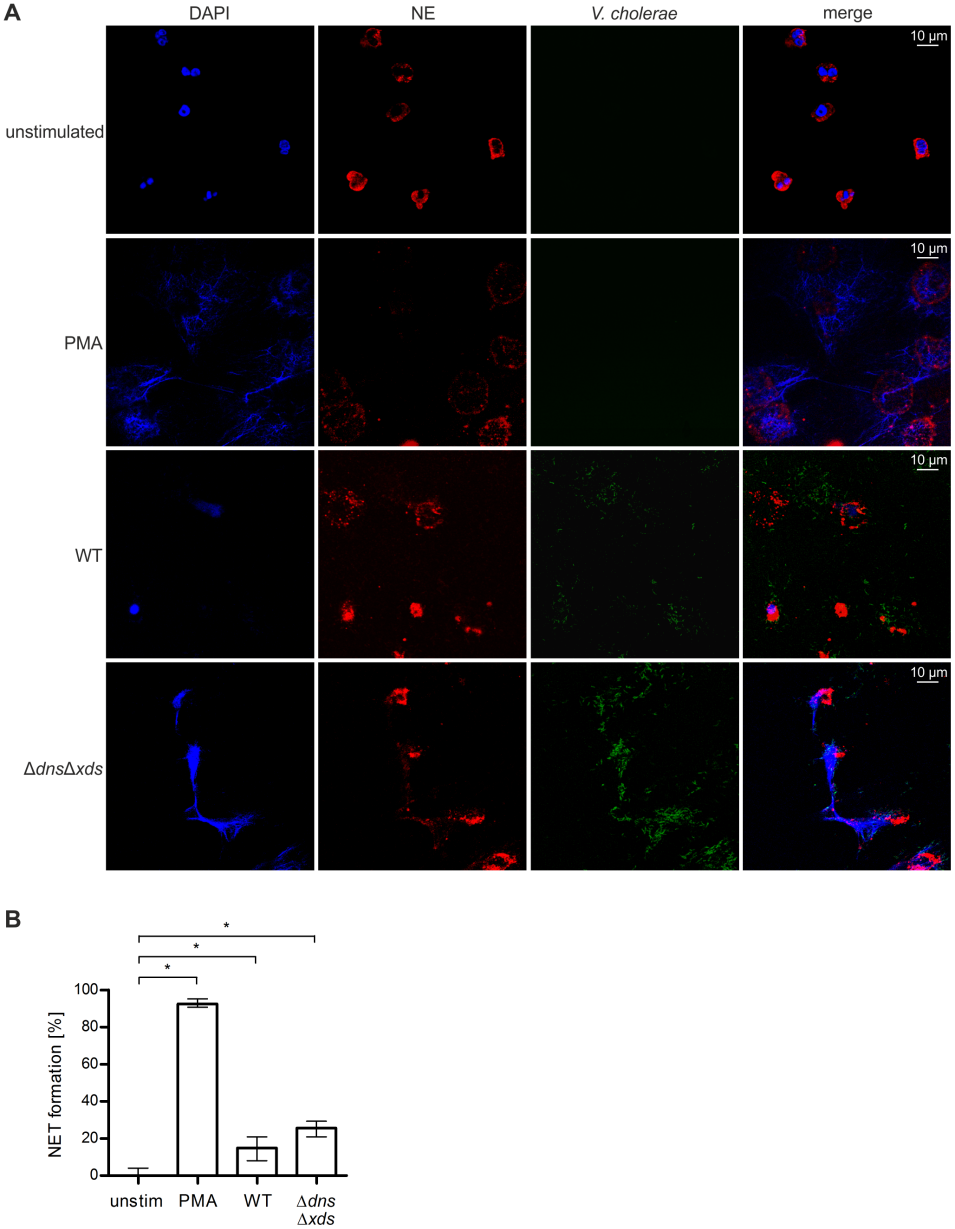
Visualization of NET formation by *V. cholerae*. Human neutrophils were stimulated for 6 h either with PMA, *V. cholerae* WT or Δ*dns*Δ*xds* mutant (MOI 40) or left untreated (unstimulated). **A**. Shown are representative immunofluorescent micrographs of DNA (DAPI, blue) and neutrophil elastase (NE, Cy3 conjugated, red) stained samples as well as *V. cholerae* expressing GFP (green). **B**. Microscopic evaluation of NET formation by human neutrophils stimulated with PMA, *V. cholerae* WT or Δ*dns*Δ*xds* mutant or left untreated (unstim). The percentage of cells undergoing NET formation was determined using ImageJ as explained in the Materials and Methods section. Shown are medians with the interquartile range. At least nine images from two independent donors were analyzed for each data set. Significant differences between the data sets are marked by asterisks (*P*<0.05; Kruskal-Wallis test followed by post-hoc Dunn's multiple comparisons).

To visualize NET formation induced by *V. cholerae* in more detail, neutrophils were stimulated with PMA, wild type, Δ*dns*Δ*xds* mutant or left unstimulated and subsequently analyzed by confocal microscopic immunofluorescence (Fig. 4A). In the unstimulated control sample intact neutrophils with lobulated nuclei and a granular staining for neutrophil elastase within the cytoplasm of cells could be observed. Micrographs of PMA stimulated neutrophils showed web-like structures of DNA with neutrophil elastase dispersed over areas larger than original cells indicating NET formation [47]. In wild type stimulated neutrophils large areas with neutrophil elastase surrounded by bacteria were visible. However, only few spots of DNA with low intensity and nearly no web-like structures could be detected. Especially, the absence of DNA in areas of elastase signal strongly suggests degradation of the DNA in NETs by the extracellular nucleases. In the case of neutrophils stimulated with Δ*dns*Δ*xds* mutant intense web-like DNA structures and neutrophil elastase signals could be observed. Consistent with the results of the degradation assay and live cell imaging, the Δ*dns*Δ*xds* is not capable to degrade DNA of the NETs. In comparison to the wild type, the Δ*dns*Δ*xds* mutant exhibits a stronger colocalization with decondensed neutrophil elastase and DNA. NET formation induced by *V. cholerae* was also microscopically quantified according to previously described methods [45], [49]. The technology is based on the determination of the DAPI DNA signal area occupied by intact cells or NETs (in µm^2^), which provides an appropriate measure to distinguish NETs from intact or non-NETotic dead neutrophils and allows a quantification of NET formation based on morphological structures (see Materials and Methods for details). Using this approach we determined that 90% of the PMA-stimulated neutrophils, 15% of wild type incubated neutrophils and 30% of the double mutant incubated neutrophils underwent NET formation after 6 h (Fig. 4B). Overall these values are quite comparable to the percentage DNA fluorescence at the 6 h time point measured by the extracellular DNA assay (Fig. 2) and therefore confirm these results. Again, lower levels of NET formation in the wild type can be explained by the degradation of extracellular DNA due to the activity of the two nucleases as observed in microscopic images (Fig. 4A). Thus, the real level of NET formation is most likely underestimated for the wild type by assays based on extracellular DNA levels.

### Extracellular nucleases of *V. cholerae* are induced by NETs

**Figure 5:**
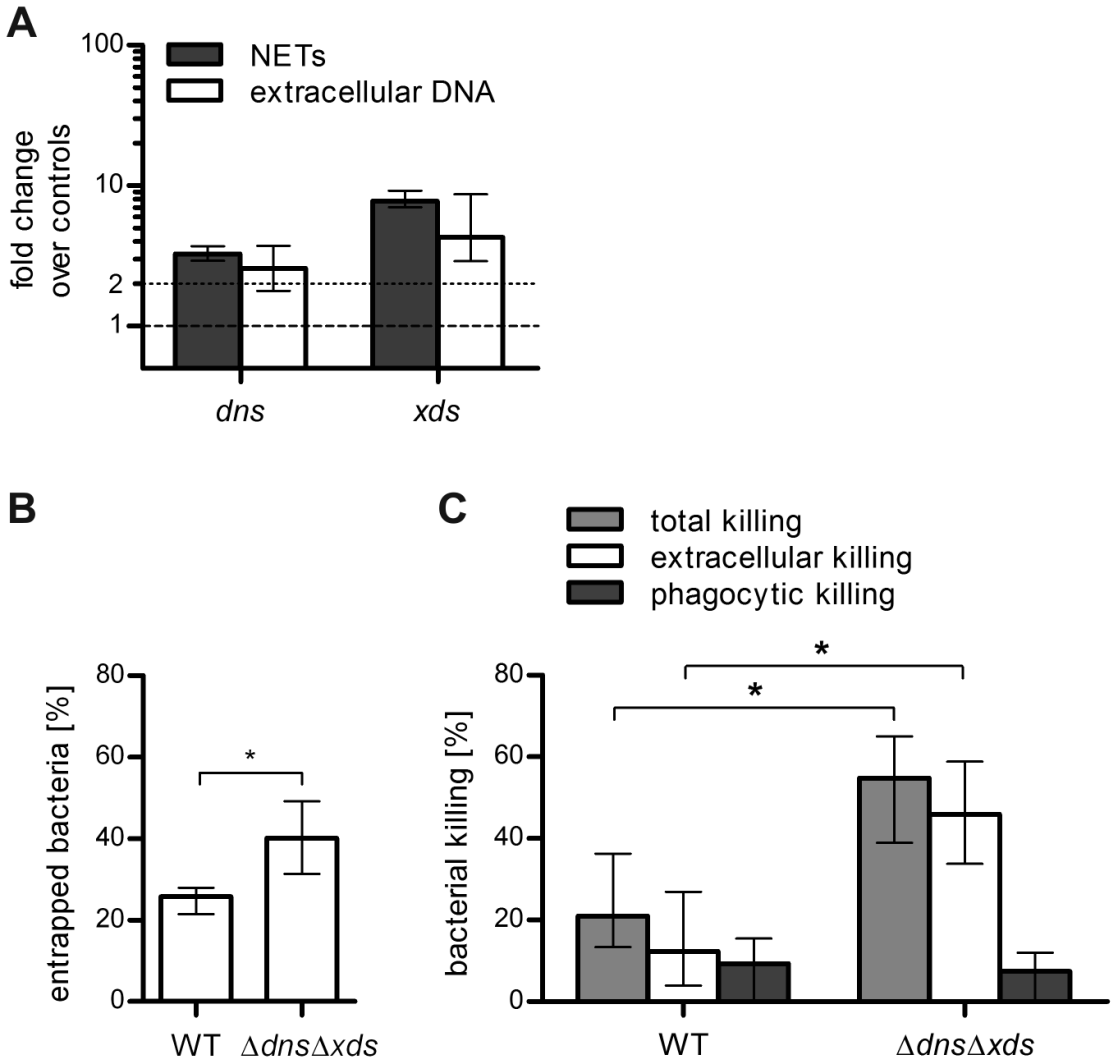
The two extracellular nucleases of *V. cholerae* facilitate survival upon contact with NETs. **A**. Induction of *dns* and *xds* upon presence of NETs (gray bars) or extracellular DNA (open bars). NET formation of human neutrophils was stimulated by PMA followed by incubation with WT *V. cholerae* (MOI 4). After incubation with NETs or extracellular DNA the bacterial RNA was extracted, reverse transcribed to cDNA and used as template for qRT-PCR analysis of the indicated genes. Expression of the analyzed genes was compared to the control condition using the same procedure but without the presence of NETs or extracellular DNA (indicated by the dashed line at 1) and normalized to the 16S rRNA. The dotted line indicates a 2-fold upregulation compared to the control conditions. The data are presented as medians with the interquartile range (n = 6). **B**. Quantitative analysis of bacterial entrapment by activated human neutrophils. NET formation was stimulated with PMA followed by incubation with the indicated *V. cholerae* strains (MOI 40). After incubation the plates were centrifuged, the supernatants were removed and the wells were carefully washed with fresh medium to remove not entrapped bacteria. Shown is the percentage of entrapped bacteria compared with the total number of bacteria in the well (100%). The data are presented as medians with the interquartile range (n = 6). Significant differences between the data sets are marked by asterisks (*P*<0.05; Mann-Whitney U Test). **C**. Quantitative analysis of bacterial killing after coincubation of PMA stimulated neutrophils with the respective *V. cholerae* strain (MOI 40). Data are presented as median percentage of killed bacteria compared with the respective control treated with DNAse I and cytochalasin D to avoid extracellular and phagocytic killing. The data are presented as medians with the interquartile range (n = 6). Significant differences between the data sets are marked by asterisks (*P*<0.05; Mann-Whitney U Test).

Noteworthy, complete degradation of NETs by *V. cholerae* wild type requires several hours (Fig. 2, 3 and 4). This might indicate, that the extracellular nucleases are not constitutively expressed, but rather induced upon contact with NETs. To determine if gene expression of nucleases is induced in presence of NETs, qRT-PCR of samples extracted from wild type *V. cholerae* incubated with PMA stimulated neutrophils was performed (Fig. 5A). Bacteria incubated in absence of neutrophils served as control condition. *V. cholerae* incubated with NETs were characterized by upregulated expression of *dns* (3-fold) and *xds* (8-fold) compared to the control condition. Interestingly, a similar induction was observed by incubating *V. cholerae* just with commercially available herring sperm DNA. Thus, *V. cholerae* induces expression of the two extracellular nucleases in presence of extracellular DNA, which is an abundant component of NETs.

### Extracellular nucleases of *V. cholerae* promote escape from NETs and enhance survival of *V. cholerae* in the presence of NETs

The colocalization of *V. cholerae* and NETs observed in Fig. 4A already provides some evidence that the Δ*dns*Δ*xds* mutant gets efficiently trapped within NETs. Indeed, NETs have been shown to trap and kill bacteria mediated through antimicrobial granule proteins decorating the NET structure and chromatin [21]. To investigate whether *V. cholerae* is ensnared in NETs, a plate based NET entrapment assay was performed. NET formation was stimulated with PMA followed by incubation with *V. cholerae* wild type or Δ*dns*Δ*xds* mutant (Fig. 5B). A significant higher amount of Δ*dns*Δ*xds* mutant bacteria was found to be entrapped compared to wild type bacteria. In concordance with the NET visualization and degradation results, the reduced entrapment rate for the wild type is most likely due to degradation of NETs by the activity of the two extracellular nucleases.

NETs not only entrap microbes and prevent spreading of the infection, but can also effectively kill bacteria due to antimicrobial effectors present at high concentrations in NETs [22]. To test whether *V. cholerae* is killed within NETs, a appropriate killing assay was conducted. Bacterial killing after coincubation of PMA stimulated neutrophils with wild type *V. cholerae* or Δ*dns*Δ*xds* mutant was measured (Fig. 5C). A significantly higher total and extracellular killing rate of Δ*dns*Δ*xds* mutant bacteria compared to wild type bacteria was observed. Noteworthy, the phagocytic killing was similar for wild type and Δ*dns*Δ*xds* mutant, whereas the increased total killing of the Δ*dns*Δ*xds* mutant predominantly resulted from increased extracellular killing representing NET-mediated killing.

## Discussion

Until now, only little attention has been drawn to the innate immune response during a *V. cholerae* infection. Naturally, the overall inflammatory response to *V. cholerae* colonization is moderate compared to that seen during infections with enteroinvasive bacterial pathogens, e.g. *Yersinia enterocolitica* or *Salmonella enterica*, which are capable of penetrating the gut epithelium of the host and cause systemic infection [50], [51]. However, emerging evidence by the recent literature indicates a substantial inflammatory response during cholera infection, which is marked by an induction of inflammatory cytokines as well as by recruitment of innate immune cells to the site of infection [10]–[12]. In agreement with these reports, we also observed a significant upregulation of inflammatory markers as well as infiltration of neutrophils into the murine gastrointestinal tract upon colonization with *V. cholerae*.

Neutrophils act as first innate immune cells, which migrate to the site of infection for containment and clearance of the pathogens. Furthermore, activated neutrophils release a variety of chemokines for activation as well as recruitment of macrophages and dendritic cells [52], [53]. Antimicrobial strategies of neutrophils range from phagocytosis of the respective microbes to the formation of NETs, which are able to bind and kill pathogens due to a high concentration of antimicrobial compounds [21], [22]. NET formation of neutrophils upon contact with Gram negative and Gram positive bacteria as well as fungi has been described [21], [54]. To withstand these attacks pathogens have evolved several mechanisms to circumvent neutrophil killing, which range from prevention of phagocytosis and intracellular survival to evasion of NETs. The later relies on extracellular nucleases degrading the chromatin fibers, which is the major structural component and scaffold of NETs. So far, this mechanism was solely described for the Gram positive bacteria, such as *S. aureus*, *S. pneumoniae* or group A *Streptococcus* causing skin or lung infections [23]–[25]. The data presented herein demonstrate that the Gram negative enteric pathogen *V. cholerae* also uses the activity of two extracellular nucleases, Dns and Xds, to efficiently degrade NETs and thereby evade entrapment and killing by NETs. The impact of this evasion mechanism is highlighted by a colonization defect of the double nuclease mutant in immunocompetent mice at 72 h. It seems reasonable that the neutrophils have to reach the colonization site first, before they can affect colonization. Accordingly, neutrophil infiltration and an increase of inflammation are already detectable at 24 h, while the impact of neutrophils on colonization requires their action on the luminal side and consequently manifests with a delay. The fact, that the observed colonization defect of the double nuclease mutant is negated in neutropenic mice corroborates the contribution of the extracellular nucleases on the evasion of NETs as part of the innate immune response against cholera. Despite intensive analyses detection of NET-like structures, defined by colocalization of MPO, histones and DAPI [21], [47], [55], in the lumen of the gastrointestinal tract failed, while the visualization of neutrophil infiltration in the epithelium of infected mice was successful. As demonstrated by this study, the wild type rapidly degrades NETs, which reduces the likelihood to detect NETs in vivo. More stable NETs might exist in double nuclease mutant infected mice, but several technical limitations have to be taken into account. To our knowledge NET visualization has so far not been achieved in the lumen of the gastrointestinal tract, which has apparent degradative activities, exhibits a pronounced auto-fluorescence and mechanical forces act on the luminal content by the peristaltic movements. Thus, NETs could rapidly loose their characteristic shape and will not be detectable above the background.

Recently, Queen and Satchell reported for a nontoxigenic (cholera toxin-deficient) *V. cholerae* strain that spread of the infection to spleen or liver within the first 6 h post infection is enhanced in neutropenic mice, while depletion of neutrophils did not affect the colonization levels in the gastrointestinal tract [19]. Based on these results, the authors hypothesized that neutrophils are required for containment of the infection to the gastrointestinal tract. Consistent with their findings, we also observed quite comparable colonization levels of a toxigenic *V. cholerae* wild type in the gastrointestinal tract in neutropenic and immunocompetent mice. In addition, Queen and Satchell demonstrated that the enhanced clearance of a multi-toxin deficient *V. cholerae* is negated in neutropenic mice, suggesting that neutrophils can impact the disease progression [19]. The colonization data obtained in the present study using the double nuclease mutant of a toxigenic strain reinforce the role of neutrophils in controlling the intestinal colonization of *V. cholerae*. In the *V. cholerae* wild type infection the impact of neutrophils on colonization levels seems to be masked by potent mechanisms of the bacterium, such as extracellular nucleases, which allow evasion from neutrophils. In addition to the secretion of extracellular nucleases, anti-inflammatory effects of the cholera toxin and cytolytic activity of accessory toxins have been described, which could also counteract the innate immune response [16], [56], [57].

Since these mechanisms of *V. cholerae* very likely act synergistically, one might only see the full impact of neutrophils by deletion of all effectors. Thus, it is quite remarkable to already observe a fitness defect of *V. cholerae* in vivo just by deletion of the two extracellular nucleases. Although the colonization level of the double nuclease mutant was significantly reduced compared to wild type, a successful colonization by the mutant was still established. Thus, the contribution of neutrophils on reduction of the overall colonization level seems to be moderate and might not affect the disease progression in the individual host to a large extend. However, rapid proliferation in the gut and leaving the host in high numbers is a key factor for transmission of cholera [6], [8]. Reduction of the total colonization level in the host by 60%, as observed for the double nuclease mutant, could consequently decrease the amount of bacteria released in the stool and might minimize reinfections and the spread of the disease. In the case of Group A *Streptococcus*, it has been demonstrated that G-actin effectively blocks bacterial nuclease activity and enhances neutrophil killing of the pathogen in vitro [23], but whether therapeutic approaches using nuclease inhibitors are applicable and can be used in the case of cholera outbreaks needs further investigation.

Noteworthy, the expression of MIP-2 and KC, representing chemokines for attraction of innate immune cells, in the host tissue was significantly higher in mice infected with double nuclease mutant compared to those infected with wild type. One explanation could be that prolonged entrapment of the double nuclease mutant in NETs allows recruitment of additional innate immune cells to the site of infection and a continuous presentation of the bacterium to the immune system, which might enhance the chemokine response. However, *V. cholerae* wild type is present at even higher numbers and consequently should also serve as a potent stimulus for the innate immune cells. Interestingly, Uchiyama et al. recently showed that group A *Streptococcus* degrade their own DNA by the activity of the Sda1 nuclease, which abrogates recognition of bacterial DNA by TLR9 and prevents TLR9-dependent cytokine release, such as IFN-α and TNF-α [58]. Indeed, we confirmed the expression of these cytokines is significantly induced in murine *Tlr4^−/−^* macrophages or human neutrophils by incubation with genomic DNA derived from *V. cholerae* compared to DNAse I-treated DNA controls (Fig. S4). Thus, degradation of *V. cholerae* DNA by the activity of the extracellular nucleases could contribute to the silencing of the innate immune system during colonization of *V. cholerae*.

Recently we hypothesized that one signal for induction of both nucleases in mature biofilms might be nutrient limitation, since both nucleases are induced under conditions of phosphate limitation [27]. As demonstrated in this study, the presence of NETs, i.e. extracellular DNA, seems to be an alternative signal for induction of the nucleases. Recently, *xds* was characterized as a gene induced at late stages of the infection [26]. Indeed, extracellular DNA as a major component of NETs seems to be a potent inducing signal for the nucleases inside the host. Ongoing research in our laboratory currently aims to elucidate the regulatory pathways and sensors involved.

As described previously Dns exhibits endonuclease activity, while Xds has been characterized as an exonuclease [27], [59], [60]. Notably, NET degradation was more affected by the deletion of *dns* compared to the deletion of *xds*. This might argue for Dns being the dominant nuclease. One explanation might be the simple fact that an endonuclease has more target sites at the DNA compared to an exonuclease requiring free ends of DNA. Given the fact that endonuclease activity creates free DNA strand ends, which can be further cleaved by exonucleases, it seems rational that Dns is the predominant nuclease. Dns in turn does not require the exonuclease activity for full functionality. The double mutant showed the most prominent effects with no measurable degradation of host DNA. Consequently, we solely used the double mutant for following assays throughout the manuscript. The absence of any detectable NET degradation activity in the double nuclease mutant strongly suggests that Dns and Xds are the only extracellular nucleases essential for NET degradation. Consistent with the effects on biofilm formation reported previously [27], it seems that Dns and Xds can at least partially compensate for each other and they may act somewhat synergistically.

As a facultative human pathogen, the lifecycle of *V. cholerae* is marked by transitions between the environmental state in the aquatic ecosystems and the in vivo state inside the gastrointestinal tract of the host. Together with the results from our previous study [27], we can now assign a dual role for the two extracellular nucleases of *V. cholerae*. On the one hand, they play a critical role in the development of a mature biofilm morphology by modulating the extracellular DNA content of the biofilm matrix and allow the utilization of DNA as a phosphate source in nutrient limited aquatic environments [27]. On the other hand, the data presented herein suggest that extracellular nucleases may facilitate the colonization fitness in vivo by liberation of the bacteria through degradation of NETs, which reduces NET mediated entrapment and killing. Similar to their role in the aquatic environment, the two nucleases of *V. cholerae* could also act as a nutrient scavenging mechanism in the host and facilitate the utilization of DNA as a nutrient source. Thus, *V. cholerae* efficiently uses the activity of the extracellular nucleases along its lifecycle to facilitate its survival in both habitats, the aquatic environment and the human host.

## Materials and Methods

### Ethics statement

This study was carried out in strict accordance with the recommendations in the Guide for the Care and Use of Laboratory Animals of the “Bundesgesetzblatt fuer die Republik Oesterreich” and the National Institutes of Health. The protocol was approved by the Committee on the Ethics of Animal Experiments of the University of Graz as well as the Austrian Federal Ministry for Science and Research BM.W-F (Permit Number: 39/53/30 ex2012/13). Mice were housed with food and water *ad libitum* and monitored under the care of full-time staff and in accordance with the rules of the Institute of Molecular Biosciences at the University of Graz. Human neutrophils were harvested from peripheral blood of healthy volunteers according to the recommendations of the local ethical committee. Fully written informed consent was provided by study participants, and all investigations were conducted according to the principles expressed in the Declaration of Helsinki.

### Bacterial strains and culture conditions

Bacterial strains and plasmids used in this study are listed in Table S1; oligonucleotides are listed in Table S2. *V. cholerae* strain AC53, a spontaneous streptomycin (Sm)-resistant derivative of the clinical isolate E7946 (O1 El Tor Ogawa) was used as wild type (WT) [61]. *E. coli* strain SM10λ*pir* was used for genetic manipulations. Unless stated otherwise, strains were inoculated at an OD_600_ of 0.05 and grown to late log phase (OD_600_ of 1) in LB medium with aeration at 37°C. Bacteria were washed 1 x in PBS, resuspended in PBS and adjusted to the respective multiplicity of infection (MOI), being the ratio of *V. cholerae* to eukaryotic cells. If required, antibiotics and other supplements were used in the following final concentrations: Sm 100 µg/ml; ampicillin (Ap) 50 µg/ml; isopropyl-β-thiogalactopyranosid (IPTG) 0.5 mM.

### Construction of in-frame deletion mutants and expression plasmids

The isolation of chromosomal DNA, PCRs, the purification of plasmids or PCR products, the construction of suicide and expression plasmids as well as the subsequent generation were carried out as described previously [27].

### Isolation of neutrophils and *Tlr4^−/−^* macrophages

Neutrophil isolation was performed as described previously [49]. Peripheral blood containing neutrophils was collected from healthy volunteer donors by venipuncture in combination with Vacuette blood collection tubes (K3 EDTA, Greiner Bio-One). Briefly, neutrophils were separated from blood by two subsequent gradient centrifugation steps (800 g, 25 min) using Histopaque 1119 (Sigma-Aldrich) and percoll (Amersham), respectively. If not stated otherwise, cells were resuspended in RPMI 1640 without phenol red supplemented with 10 mM Hepes.

Macrophages were isolated from the peritoneal cavitiy from *Tlr4^−/−^* mice 72 h after injection of 2 ml Brewer thioglycollate medium (3% (w/v)) as previously described [62]. *Tlr4^−/−^* mice were kindly provided by the Institute of Experimental and Clinical Pharmacology (Medical University of Graz) [63]. Harvested *Tlr4^−/−^* macrophages were washed once and resuspended in RPMI-1640 containing 10% heat-inactivated fetal bovine serum. *Tlr4^−/−^* macrophages (5×10^5^) were seeded into a 24 well plate and incubated for 24 h before fresh medium with genomic bacterial DNA was added.

### ROS quantification

ROS production of human neutrophils was measured by a luminometric assay as described previously [49]. Briefly, 5×10^4^ cells per well were seeded in a white 96 well plate. After addition of 50 µM luminol and 1.2 U/ml horseradish peroxidase, NET formation was stimulated either with 100 nM PMA, the indicated *V. cholerae* strain (MOI 4 or 40) or left untreated. The chemiluminescence resulting from ROS production was measured every 3 min for a period of 6 h in a Tecan Infinite 200 plate reader at 37°C and 5% CO_2_. The ROS amounts are presented integral of the area under the curve or as relative light units (RLU) for ROS dynamics.

### Extracellular DNA fluorescence assay

Presence of extracellular DNA was measured as described previously [45], [49]. Briefly, 5×10^4^ cells per well were seeded in a black 96 well plate. After addition of Sytox Green (2.5 µM), neutrophils were stimulated either with 100 nM PMA, the indicated *V. cholerae* strain (MOI 4 and 40), left untreated or lysed with 1% Triton X-100 as a 100% lysis control. Fluorescence was monitored every 10 min in a plate-based fluorescence spectrometer (Fluostar, Omega, BMG), for a time period of 14 h, under cell culture conditions (37°C, 5% CO_2_). In some wells DNAse I (100 U/ml) was added after 6 h to PMA or Δ*dns*Δ*xds* mutant stimulated neutrophils as a control for DNA degradation. Percent NET formation was calculated as percentage of lysis control. Data were presented as percent DNA fluorescence.

### Live cell microscopy

For live cell microscopy 10^5^ neutrophils were seeded per chamber (dish diameter: 35 mm; microwell diameter: 14 mm; coverglass: 0.16–0.19 mm; MatTek Corp.) and NET formation was stimulated by addition of the respective *V. cholerae* strain (MOI 4) in presence of the cell impermeant fluorescent DNA dye Sytox Green. Live cell imaging was performed and the movies were recorded by Nikon Eclipse Ti-E, using appropriate filters and a Splan Fluor, ELWD 40× objective (Nikon). The movies and images were analyzed by Nis-Elements AR version 3.2.0 software.

### Degradation assay

The degradation assay was performed similar to the DNA fluorescence assay described above, with some modifications. Neutrophils were stimulated with PMA (100 nM) prior the addition of *V. cholerae* to induce a uniformly high level of NET formation. After 6 h, the indicated *V. cholerae* strain (MOI 40) was added and changes in the fluorescence were monitored every 10 min in a plate-based fluorescence spectrometer (Fluostar, Omega, BMG) for another 8 h to allow detection of NET degradation.

### Immunostaining of neutrophils and microscopy

Immunostaining of neutrophils was performed essentially as described previously [49]. Briefly, 10^5^ neutrophils per well were seeded on glass cover slips coated with 0.01% poly-L-lysine in a 24 well plate and stimulated either with PMA (100 nM), the indicated GFP expressing *V. cholerae* strain (MOI 40), or left untreated for 6 h at 37°C and 5% CO_2_. Cell were fixed with paraformaldehyde (2%), blocked (3% cold water fish gelatin, 5% fetal calf serum, 1% bovine serum albumin, and 0.25% Tween 20 in PBS), incubated with the primary antibody anti-neutrophil elastase (5 µg/ml, #BM382, Acris) in combination with a secondary antibody conjugated to Cy3 (Jackson Immuno research) and stained with DAPI (4 µg/ml) to detect DNA. Specimens were mounted in Mowiol 4–88 and microscopy was performed using a Leica HCX PL Apo 40× oil immersion objective (NA 1.25) on a confocal microscope (Leica SP2, Leica Microsystems). For visualization the Leica LAF software was used.

### Microscopic quantification of NET formation

To quantify NET formation microscopically a similar method was used as previously described [45], [49]. At least 9 random images and not less than 300 neutrophils for each condition were analyzed using the DAPI DNA signal. The ImageJ 1.46 software was used to calculate pixel areas of the DAPI signal by adjusting the threshold above background. Calculated pixel areas were converted to µm^2^. Particles covering an area less than 45 µm^2^ were excluded from analysis. The average diameter of human neutrophils is 10 µm, therefore the area of neutrophils in an unstimulated stage is approximately 80 µm^2^. Signals exceeding 100 µm^2^ were considered as neutrophils undergoing NET formation, because they were larger than the whole intact cell area, representing either released NETs or cells with decondensed nuclei, which is an essential step prior to NET release. The percentage of NET formation was calculated per image as (neutrophils undergoing NET formation/total number of neutrophils)×100.

### NET entrapment assay

A modified version of the assay described by Berends et al. was used to determine the bacterial NET entrapment [24]. Briefly, 5×10^4^ neutrophils per well were seeded in a 96 well plate and stimulated for 6 h with 100 nM PMA (37°C, 5% CO_2_) to induce NET formation, followed by addition of the respective *V. cholerae* strain (MOI 40). The plate was centrifuged 10 min at 800 g to bring the bacteria in contact with the previously formed NETs. After incubation (10 h, 37°C, 5% CO_2_) the supernatants were carefully aspirated and plated for CFU counting. The wells were gently washed with prewarmed medium to remove residual bacteria not entrapped within the NETs. Subsequently prewarmed medium containing DNAse I (100 U/ml) was added and incubated for 15 min at 37°C to dissolve the NETs and release the entrapped bacteria. This fraction was also plated for CFU counting. The percentage of entrapped bacteria was calculated as (entrapped bacteria/total number of bacteria) ×100. The total number of bacteria in the well represents the sum of bacteria in the supernatant and entrapped bacteria.

### NET killing assay

The NET killing assay was essentially performed as previously published using a 96 well plate assay [64]. Briefly, 10^6^ neutrophils per well were seeded in a 96 well plate. Neutrophils were stimulated with PMA (100 nM) and incubated with the indicated *V. cholerae* strain (MOI 40). Noteworthy, *V. cholerae* used in this assay were grown in presence of extracellular DNA to allow induction of nucleases. In some wells DNAse I (100 U/ml) was directly added as a control to allow degradation of NETs and inhibit NET-mediated killing. Furthermore, phagocytic killing was inhibited in some wells by the addition of cytochalasin D (100 µg/ml) 15 min prior to the addition of PMA and bacteria. The plate was centrifuged 10 min at 800 g, to bring bacteria in contact with neutrophils or NETs and incubated 4 h at 37°C and 5% CO_2_. Subsequently, all wells having not received DNAse I, were treated with DNAse I for 15 min (37°C, 5% CO_2_) to degrade the NETs as well, which results in release of all remaining bacteria for total CFU count in the respective wells as suggested by Menegazzi et al. [65]. Finally, Triton X-100 (0.01%) was added to all wells, the well content was passed three times through a 25 gauge needle to disrupt neutrophils as well as clumped NETs and plated for CFU counting. According to Young et al. [64], neutrophils incapable of killing seemed to enhance bacterial recovery. Hence, “zero killing” was determined by the CFU recovered from control wells consisting of neutrophils treated with DNAse I and cytochalasin D to inhibit NET-mediated and phagocytic killing, respectively. NET-mediated killing was calculated by subtracting the extent of killing in the presence of DNAse I (i.e. phagocytic killing) from the extent of killing in absence of DNAse I and cytochalasin D (total killing).

### In vivo experiments

For evaluation of the in vivo colonization fitness we used the previously established adult mouse model by Nygren et al., which results in a stable colonization of the cecum and colon [28]. Briefly, eight to ten weeks old C57BL/6 immunocompetent or neutropenic mice were given Sm (5 mg/ml) in their drinking water, two days prior to infection and kept on Sm-water (0.2 mg/ml) after inoculation with the respective *V. cholerae* strain (7×10^9^ to 10^10^ CFU per mouse) or left uninfected for mock-inoculated controls. At 24 h or 72 h post infection mice were euthanized and the ceca and colons removed. Relatively large amounts of fluid can accumulate in the ceca of infected mice, which can be released together with bacteria as soon as the cecum is harvested. Thus, cecum samples are not ideal candidates for reproducible quantification of the colonization levels and were consequently only used for histological analysis. Ceca were fixed in 4% buffered formaldehyde, embedded in paraffin, cut in sections (5 µm) and used for histological evaluation. The proximal 1 cm of the ascending colon was used for RNA preparation to determine the inflammatory response. The residual colon was homogenized in LB medium and plated for CFU counting.

To induce neutropenia in mice the monoclonal antibody 1A8/Anti-Ly6G mAb (BioXCell) was used as described previously [19], [46]. 16 h prior to oral inoculation with *V. cholerae*, mice were injected intraperitoneally with 0.8 mg of 1A8 or not injected in case of the immunocompetent mice. A decrease of neutrophils by at least 2-fold compared to immunocompetent mice was considered as neutropenia, which was confirmed by analyses of peripheral blood smears by trained medical staff at the time point of inoculation as well as 72 h post infection.

### Immunostaining of mouse tissue

For immunostaining, specimens were processed similarly as described previously [66]. Briefly, samples were deparaffinized, rehydrated in decreasing concentrations of EtOH, and subjected to antigen retrieval by cooking in 10 mM citrate buffer, pH 6.0, for 10 min. Specimens were blocked with 2% BSA and 36 µl/ml mouse Ig blocking reagent (Vector Laboratories) in PBS/0.1% Triton for 1 h at room temperature. For visualization of neutrophils, a primary antibody directed against MPO (A0398, Dako) diluted in blocking solution was applied over night at 4°C. Additionally, chromatin was detected using a primary antibody directed against histone H1 (clone AE-4, Acris Antibodies). Primary antibodies were detected with Alexa Fluor 568- and 488-conjugated secondary antibodies (Life Technologies) diluted in 2% BSA in PBS/0.1% Triton, respectively. DNA was visualized with DAPI (Life Technologies) and slides were mounted with fluorescence mounting medium (Dako). Images were captured with a C1 confocal microscope (Nikon Instruments) at 60× magnification and are presented as maximum intensity projections from Z-stacks.

### Preparation of RNA and qRT-PCR

To measure gene expression of the two extracellular nucleases of *V. cholerae* in presence of NETs, 10^6^ neutrophils per well were seeded in a 24 well plate, stimulated with PMA (100 nM, 4 h), followed by incubation for 6 h with *V. cholerae* wild type strain (MOI 40). In case of DNA as a stimulus, *V. cholerae* wild type strain was incubated for 6 h with 2.5 mg/ml herring sperm DNA (Sigma). *V. cholerae* incubated in absence of neutrophils or extracellular DNA served as a control. Bacterial RNA was extracted using the RNeasy Mini Kit (Qiagen) and chromosomal DNA was removed using RQ1 RNAse-Free DNase (Promega) according to the manufacturer's protocol.

To measure the induction of inflammatory gene expression upon *V. cholerae* infection, the proximal 1 cm of the ascending colon was collected and homogenized with a Power Lyzer24 (Mobio) in 1 ml Trizol. For DNA-mediated induction of gene expression, *Tlr4* ^−/−^ murine macrophages (isolation see above) were stimulated with *V. cholerae* wild type genomic DNA (2.5 µg/ml) for 12 h or human neutrophils (10^6^) were stimulated with *V. cholerae* Δ*msbB* genomic DNA (2.5 µg/ml) for 8 h using a 24 well plate, respectively. Incubation with DNAse I digested wild type or Δ*msbB* genomic DNA (2.5 µg/ml) served as control conditions. After the stimulation time *Tlr4* ^−/−^ macrophages or human neutrophils were resuspended in Trizol, RNA was extracted using chloroform extraction and precipitated with isopropanol.

The cDNA synthesis was performed with an iScript Select cDNA Synthesis Kit (Bio-Rad) using 200 ng bacterial RNA or 1 µg mouse/human RNA. Quantitative RT-PCR was performed with SYBR GreenER qPCR SuperMix for ABI PRISM instrument (Invitrogen) utilizing a Rotor-Gene 600 and Rotor-Gene 600 Series Software 1.7 (GenXpress) according to the manufacturer's instructions. Each reaction contained primers (400 ng) and template (10 ng for bacterial cDNA as well as 50 ng for mouse and human cDNA) and was tested in triplicate. The sequences of the primers used for qRT-PCR are listed in Table S2, labeled as x_fw and x_rv, in which x stands for the respective gene. For each sample, the mean cycle threshold of the test transcript was normalized to the housekeeping gene 16S rRNA (bacterial samples) or 36B4 (mouse and human samples), also known as RPLP, and to one randomly selected control sample.

### Statistical analysis

Unless stated otherwise the data is presented as the median with interquartile range. Data were analyzed using the Mann-Whitney U test for single comparisons or Kruskal-Wallis test followed by post-hoc Dunn's multiple comparisons. Differences were considered significant for *P* values of <0.05. For all statistical analyses the GraphPad Prism 4.0a software was used.

## Supporting Information

Figure S1 **Visualization of neutrophil infiltration in the cecal mucosa of mice colonized with** ***V. cholerae*** **.** Shown are representative images (magnification 600×) of tissue sections of mouse ceca colonized for 24 h with *V. cholerae* WT, *ΔdnsΔxds* mutant or left uninfected as mock-inoculated control. For histological evaluation the ceca were fixed in 4% formaldehyde and embedded in paraffin. Tissue sections (5 µm) were stained with hematoxylin/eosin following standard protocols for enhanced visualization. Arrows indicate neutrophils within the epithelium.
(TIF)

Figure S2 **Controls for ROS production and ROS dynamics.** **A**. Human neutrophils were stimulated with PMA or left untreated (unstim) and the ROS production was measured by a luminometric assay. The y-axis shows the area under the curve representing the ROS production over 6 h. Shown are medians of at least six measurements out of three independent donors. The error bars represent the interquartile range. **B and C**. Representative ROS production by human neutrophils incubated with the indicated *V. cholerae* strains and MOI was measured by a luminometric assay. The y-axis shows the relative light units (RLU) representing the temporal ROS production. Shown are medians of at least three measurements. The error bars represent the interquartile range.
(TIF)

Figure S3 **Analysis of DNA release by neutrophils.** **A–D**. Shown is the temporal DNA release of neutrophils stimulated with the indicated *V. cholerae* strain and MOI. Staining of DNA by the cell impermeant fluorescent DNA dye Sytox green was measured in 10 min intervals. Values are presented as percentage of DNA fluorescence compared with the Triton ×100 lysis control (100%) indicating NET formation, respectively. Shown are medians of at least six measurements out of two independent donors.
(TIF)

Figure S4 **Stimulation of** ***Tlr4^−/−^*** **macrophages and human neutrophils by addition of** ***V. cholerae*** **DNA.** DNA-mediated induction of TNF-α and IFN-α gene expression was determined by qRT-PCR. *Tlr4^−/−^* macrophages (**A**) or human neutrophils (**B**) were stimulated with genomic DNA (2.5 µg/ml) derived from *V. cholerae* WT or *ΔmsbB* mutant, respectively. To avoid side effects by LPS contamination of the genomic DNA preparation we either used *Tlr4^−/−^* murine macrophages not able to get stimulated by LPS or in case of human neutrophils genomic DNA derived from a *V. cholerae msbB*-mutant with underacylated LPS, which is consequently only a weak stimulus for TLR4 activation as previously demonstrated [67], [68]. Gene expression was normalized to the housekeeping gene 36B4. Shown are median gene expression levels compared to DNAse I-treated genomic DNA controls (indicated by the dashed line at 1) for each data set (n≥5). Thus, any residual contamination (proteins or LPS) present in the DNA preparation, which could cause an upregulation of inflammatory markers, should affect *Tlr4^−/−^* macrophages and human neutrophils in the test and control condition in a similar way. The error bars indicate the interquartile range. The dotted line indicates a 2-fold upregulation compared to the controls.
(TIF)

Movie S1 **Human neutrophils stimulated with** ***V. cholerae*** **WT (MOI 4).** Time-lapse movie in presence of the cell impermeant fluorescent DNA dye Sytox green. Live cell images were taken every 5 minutes, the time is indicated on the upper left. Individual images of representative time points are shown in Fig. 3B.
(AVI)

Movie S2 **Human neutrophils stimulated with Δ** ***dns*** **Δ** ***xds*** **mutant (MOI 4).** Time-lapse movie in presence of the cell impermeant fluorescent DNA dye Sytox green. Live cell images were taken every 5 minutes, the time is indicated on the upper left. Individual images of representative time points are shown in Fig. 3B.
(AVI)

Table S1 **Strains and plasmids used in this study.**
(DOC)

Table S2 **Oligonucleotides used for qRT-PCR and deletion mutagenesis.**
(DOC)
