# Supplementary figures and images for: Vibrio cholerae Evades Neutrophil Extracellular Traps by the Activity of Two Extracellular Nucleases
Source: PLoS Pathog. 2013 Sep 5;9(9):e1003614. doi: 10.1371/journal.ppat.1003614 (PMC3764145; doi:10.1371/journal.ppat.1003614)

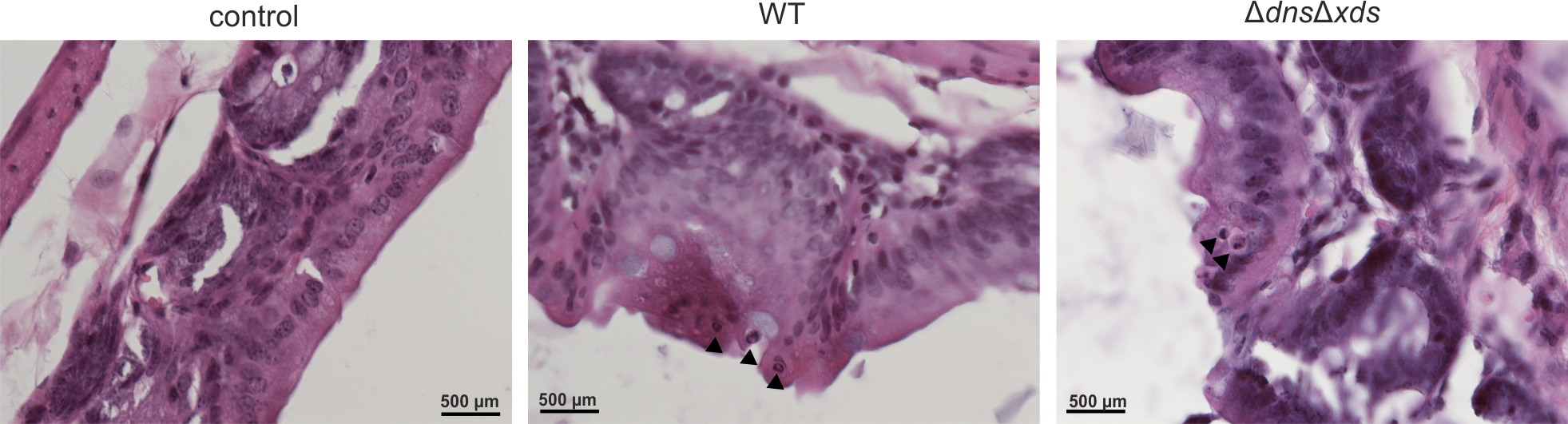

Supplement: Figure S1 — Visualization of neutrophil infiltration in the cecal mucosa of mice colonized with V. cholerae . Shown are representative images (magnification 600×) of tissue sections of mouse ceca colonized for 24 h with V. cholerae WT, ΔdnsΔxds mutant or left uninfected as mock-inoculated control. For histological evaluation the ceca were fixed in 4% formaldehyde and embedded in paraffin. Tissue sections (5 µm) were stained with hematoxylin/eosin following standard protocols for enhanced visualization. Arrows indicate neutrophils within the epithelium. (TIF) [file ppat.1003614.s001.tif]

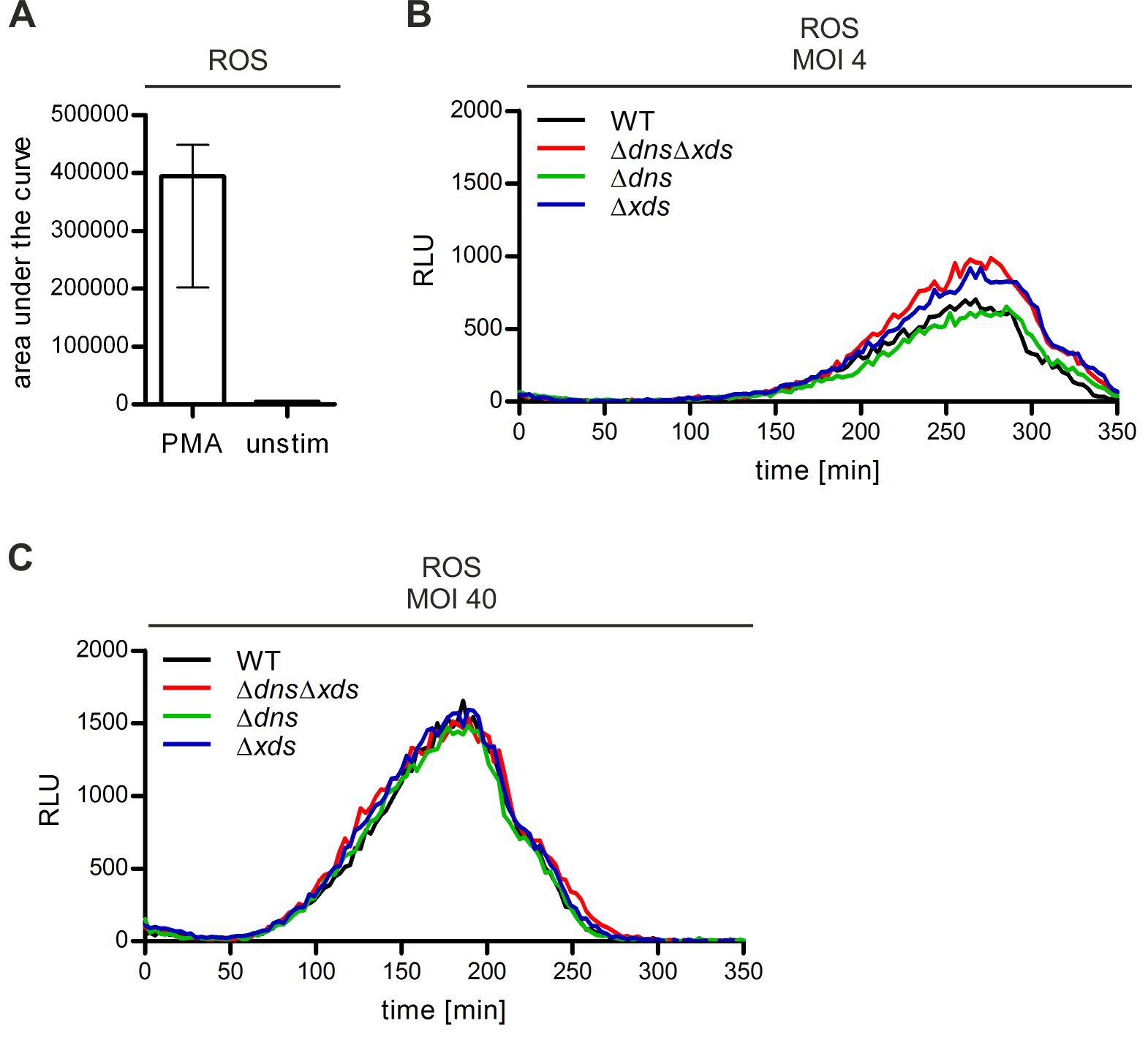

Supplement: Figure S2 — Controls for ROS production and ROS dynamics. A. Human neutrophils were stimulated with PMA or left untreated (unstim) and the ROS production was measured by a luminometric assay. The y-axis shows the area under the curve representing the ROS production over 6 h. Shown are medians of at least six measurements out of three independent donors. The error bars represent the interquartile range. B and C. Representative ROS production by human neutrophils incubated with the indicated V. cholerae strains and MOI was measured by a luminometric assay. The y-axis shows the relative light units (RLU) representing the temporal ROS production. Shown are medians of at least three measurements. The error bars represent the interquartile range. (TIF) [file ppat.1003614.s002.tif]

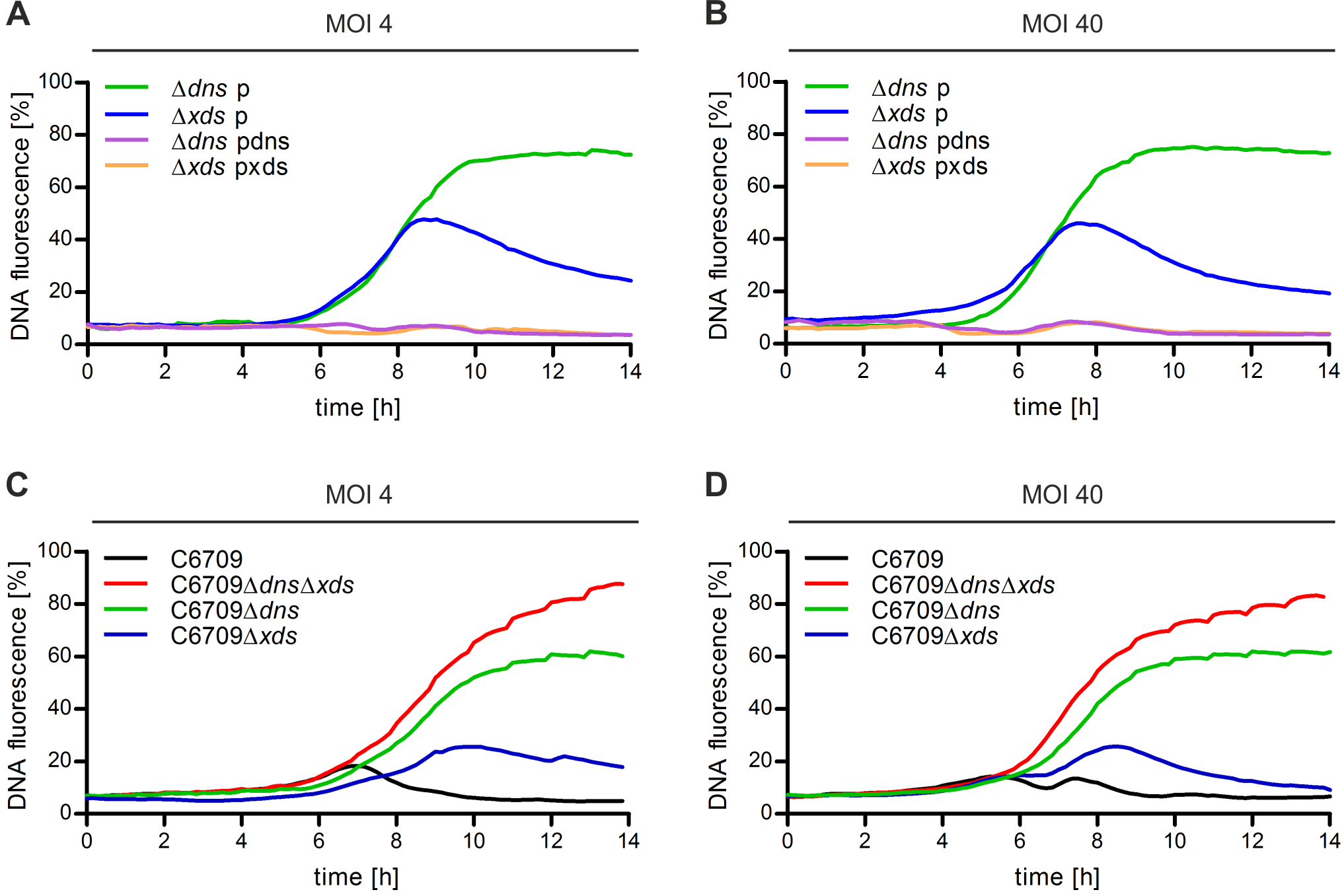

Supplement: Figure S3 — Analysis of DNA release by neutrophils. A–D. Shown is the temporal DNA release of neutrophils stimulated with the indicated V. cholerae strain and MOI. Staining of DNA by the cell impermeant fluorescent DNA dye Sytox green was measured in 10 min intervals. Values are presented as percentage of DNA fluorescence compared with the Triton ×100 lysis control (100%) indicating NET formation, respectively. Shown are medians of at least six measurements out of two independent donors. (TIF) [file ppat.1003614.s003.tif]

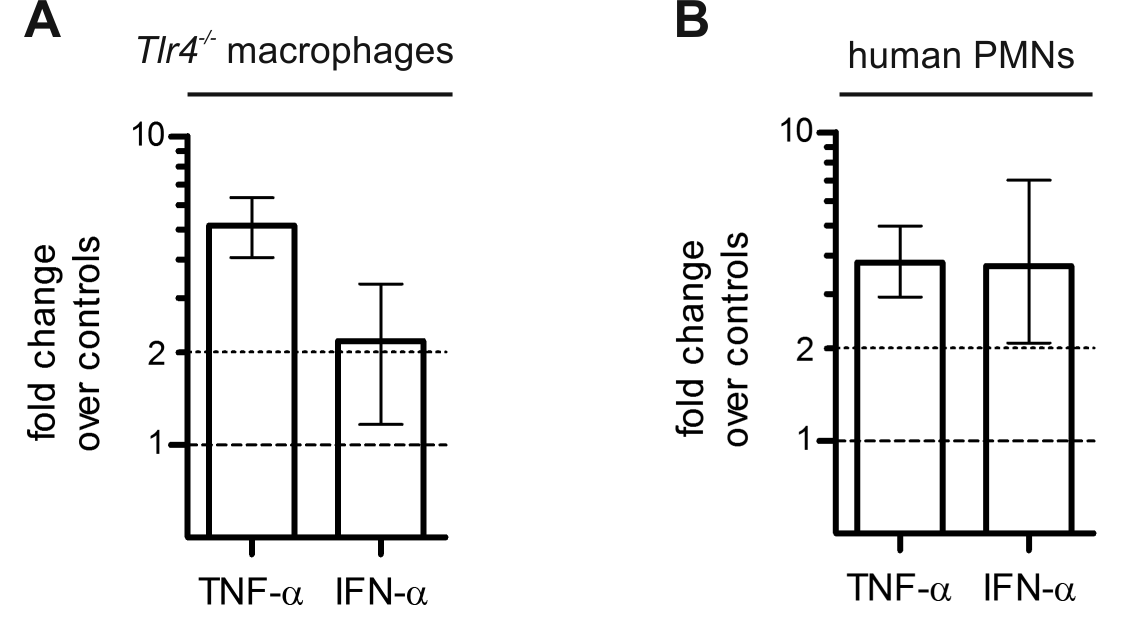

Supplement: Figure S4 — Stimulation of Tlr4−/− macrophages and human neutrophils by addition of V. cholerae DNA. DNA-mediated induction of TNF-α and IFN-α gene expression was determined by qRT-PCR. Tlr4−/− macrophages (A) or human neutrophils (B) were stimulated with genomic DNA (2.5 µg/ml) derived from V. cholerae WT or ΔmsbB mutant, respectively. To avoid side effects by LPS contamination of the genomic DNA preparation we either used Tlr4−/− murine macrophages not able to get stimulated by LPS or in case of human neutrophils genomic DNA derived from a V. cholerae msbB-mutant with underacylated LPS, which is consequently only a weak stimulus for TLR4 activation as previously demonstrated [67], [68]. Gene expression was normalized to the housekeeping gene 36B4. Shown are median gene expression levels compared to DNAse I-treated genomic DNA controls (indicated by the dashed line at 1) for each data set (n≥5). Thus, any residual contamination (proteins or LPS) present in the DNA preparation, which could cause an upregulation of inflammatory markers, should affect Tlr4−/− macrophages and human neutrophils in the test and control condition in a similar way. The error bars indicate the interquartile range. The dotted line indicates a 2-fold upregulation compared to the controls. (TIF) [file ppat.1003614.s004.tif]
