# Supplementary material for: Vibrio cholerae Evades Neutrophil Extracellular Traps by the Activity of Two Extracellular Nucleases
Source: PLoS Pathog. 2013 Sep 5;9(9):e1003614. doi: 10.1371/journal.ppat.1003614 (PMC3764145; doi:10.1371/journal.ppat.1003614)
Supplement: Table S1 — Strains and plasmids used in this study. (DOC) [file ppat.1003614.s007.doc]

Table S1: Strains and plasmids used in this study

| **Strain/plasmid** | **Description** | **Reference^1^** |
| --- | --- | --- |
| *E. coli* |  |  |
| SM10λ*pir* | *thi thr leu tonA lacY supE recA*::RPA-2-Te::Mu *λpir*R6K, Km^r^ | [1] |
| *V. cholerae* |  |  |
| WT | wild type *V. cholerae* strain AC53; spontaneous Sm^r^ isolate of E7946 (O1; biotype: El Tor; serotype: Ogawa); clinical isolate from Bahrain 1978; Sm^r^; | [2] |
| Δ*dns* | Deletion of *dns* in AC53, Sm^r^ | This study |
| Δ*xds* | Deletion of *xds* in AC53, Sm^r^ | This study |
| Δ*dns*Δ*xds* | Deletion of *dns* and *xds* in AC53, Sm^r^ | This study |
| Δ*dns*Δ*xds*Δ*hlyA* | Deletion of *dns*, *xds* and *hlyA* in AC53, Sm^r^ | This study |
| ∆*msbB* | Deletion of *msbB* in AC53, Sm^r^ | [3] |
| C6709 | wild type *V. cholerae* strain C6709 (O1; biotype: El Tor; serotype: Inaba); spontaneous Sm^r^; clinical isolate; Peru 1991; Sm^r^; | [4] |
| C6709Δ*dns* | Deletion of *dns* in C6709, Sm^r^ | [5] |
| C6709Δ*xds* | Deletion of *xds* in C6709, Sm^r^ | [5] |
| C6709Δ*dns*Δ*xds* | Deletion of *dns* and *xds* in C6709, Sm^r^ | [5] |
| Plasmids |  |  |
| pCVD442 | *oriR6K mobRP4* *sacB*, Ap^r^ | [6] |
| p | pMMB67EH, IncQ broad-host-range low-copy-number cloning vector, IPTG inducible, Ap^r^ | [7] |
| pCVD442Δdns | pCVD442::Δ*dns*, Ap^r^ | [5] |
| pCVD442ΔhlyA | pCVD442::Δ*hlyA*, Ap^r^ | This study |
| pCVD442Δxds | pCVD442::Δ*xds*, Ap^r^ | [5] |
| pdns | *dns* of *V. cholerae* in pMMB, Ap^r^ | [5] |
| pxds | *xds* of *V. cholerae* in pMMB, Ap^r^ | [5] |

^1^References:

1. Miller VL, Mekalanos JJ (1988) A novel suicide vector and its use in construction of insertion mutations: osmoregulation of outer membrane proteins and virulence determinants in *Vibrio cholerae* requires *toxR*. J Bacteriol 170: 2575-2583.

2. Miller VL, DiRita VJ, Mekalanos JJ (1989) Identification of *toxS*, a regulatory gene whose product enhances *toxR*-mediated activation of the cholera toxin promoter. J Bacteriol 171: 1288-1293.

3. Leitner DR, Feichter S, Schild-Prufert K, Rechberger GN, Reidl J, et al. (2013) Lipopolysaccharide modifications of a cholera vaccine candidate based on outer membrane vesicles reduce endotoxicity and reveal the major protective antigen. Infect Immun 81: 2379-2393.

4. Roberts A, Pearson GD, Mekalanos JJ (1992) Cholera vaccines strains derived from a 1991 Peruvian isolate of *Vibrio cholerae* and other El Tor strains, p. 43-47. In: Proceedings of the 28th Joint Conference, U.S.-Japan Cooperative Medical Science Program on Cholera and Related Diarrheal Diseases. 20-21 July, 1992, Tokyo, Japan.

5. Seper A, Fengler VH, Roier S, Wolinski H, Kohlwein SD, et al. (2011) Extracellular nucleases and extracellular DNA play important roles in *Vibrio cholerae* biofilm formation. Mol Microbiol 82: 1015-1037.

6. Donnenberg MS, Kaper JB (1991) Construction of an *eae* deletion mutant of enteropathogenic *Escherichia coli* by using a positive-selection suicide vector. Infect Immun 59: 4310-4317.

7. Morales VM, Backman A, Bagdasarian M (1991) A series of wide-host-range low-copy-number vectors that allow direct screening for recombinants. Gene 97: 39-47.
