# Supplementary material for: Vibrio cholerae Evades Neutrophil Extracellular Traps by the Activity of Two Extracellular Nucleases
Source: PLoS Pathog. 2013 Sep 5;9(9):e1003614. doi: 10.1371/journal.ppat.1003614 (PMC3764145; doi:10.1371/journal.ppat.1003614)
Supplement: Table S2 — Oligonucleotides used for qRT-PCR and deletion mutagenesis. (DOC) [file ppat.1003614.s008.doc]

**Table S2: Oligonucleotides used for qRT-PCR and deletion mutagenesis**

| **Oligonucleotides** | **Sequence (5’- 3’)** |
| --- | --- |
| hlyA_XbaI_1 | AAATCTAGAAGCGGCTACTGTGTCG |
| hlyA_BamHI_2 | AAAGGATCCTGGCATATAAACCTCACTGACTCT |
| hlyA_BamHI_3 | TTTGGATCCTAAAAAGAGACCGTCAACATAGCC |
| hlyA_SacI_4 | AAAGAGCTCGAGGATCTGGTTGGCTT |
| dns_fw | ATTGAATGGGAACATGTGGTGC |
| dns_rv | GACTGAAACTGAAGTTGGAGCG |
| xds_fw | CTCCCTATATCCAAGGCTATCCC |
| xds_rv | GTAAACACGAACAGCCCTTCAG |
| 16S rRNA_fw | AGGGAGGAAGGTGGTTAAGT^a^ |
| 16S rRNA_rv | CGCTACACCTGAAATTCTACCC^a^ |
| MIP-2_fw | CCAACCACCAGGCTACAGG |
| MIP-2_rv | GCGTCACACTCAAGCTCT |
| KC_fw | CAATGAGCTGCGCTGTCAGTG^b^ |
| KC_rv | CTTGGGGACACCTTTTAGCATC^b^ |
| IL-6_fw | GAGGATACCACTCCCAACAGACC^c^ |
| IL-6_rv | AAGTGCATCATCGTTGTTCATACA^c^ |
| 36B4_fw | GCTTCATTGTGGGAGCAGACA^c^ |
| 36B4_rv | CATGGTGTTCTTGCCCATCAG^c^ |
| TNF-α_fw | CATCTTCTCAAAATTCGAGTGACAA ^c^ |
| TNF-α_rv | TGGGAGTAGACAAGGTACAACCC ^c^ |
| IFN-α_fw | TCTGATGCAGCAGGTGGG^e^ |
| IFN-α_rv | AGGGCTCTCCAGACTTCTGCTCTG^e^ |
| h_TNF-α_fw^d^ | GGAGAAGGGTGACCGACTCA^f^ |
| h_TNF-α_rv^d^ | TGCCCAGACTCGGCAAAG^f^ |
| h_IFN-α_fw^d^ | GTGAGGAAATACTTCCAAAGAATCAC^e^ |
| h_IFN-α_rv^d^ | TCTCATGATTTCTGCTCTGACAA^e^ |

^a^Oligonucleotides for 16S rRNA are according to [1]

^b^Oligonucleotides for KC are according to [2]

^c^Oligonucleotides for 36B4, IL-6 and TNF-α are according to [3]

^d^Oligonucleotides for human samples

^e^Oligonucleotides for h_TNF-α are according to [4]

^f^Oligonucleotides for h_IFN-α are according to [5]

restriction sites are underlined

**References:**

1. Fengler VH, Boritsch EC, Tutz S, Seper A, Ebner H, et al. (2012) Disulfide bond formation and ToxR activity in *Vibrio cholerae*. PLoS One 7: e47756.

2. Park SW, Chen SW, Kim M, Brown KM, Kolls JK, et al. (2010) Cytokines induce small intestine and liver injury after renal ischemia or nephrectomy. Lab Invest 91: 63-84.

3. Leitner DR, Feichter S, Schild-Prufert K, Rechberger GN, Reidl J, et al. (2013) Lipopolysaccharide modifications of a cholera vaccine candidate based on outer membrane vesicles reduce endotoxicity and reveal the major protective antigen. Infect Immun 81: 2379-2393.

4. Yeretssian G, Correa RG, Doiron K, Fitzgerald P, Dillon CP, et al. (2011) Non-apoptotic role of BID in inflammation and innate immunity. Nature 474: 96-99.

5. Gautier G, Humbert M, Deauvieau F, Scuiller M, Hiscott J, et al. (2005) A type I interferon autocrine-paracrine loop is involved in Toll-like receptor-induced interleukin-12p70 secretion by dendritic cells. J Exp Med 201: 1435-1446.
